# Monitoring and Visualization of Crystallization Processes Using Electrical Resistance Tomography: CaCO_3_ and Sucrose Crystallization Case Studies

**DOI:** 10.3390/s22124431

**Published:** 2022-06-11

**Authors:** Guruprasad Rao, Soheil Aghajanian, Yuchong Zhang, Lidia Jackowska-Strumiłło, Tuomas Koiranen, Morten Fjeld

**Affiliations:** 1 Institute of Applied Computer Sciences, Lodz University of Technology, 90-924 Lodz, Poland; guruprasad.rao@dokt.p.lodz.pl; 2 School of Engineering Science, LUT University, Yliopistonkatu 34, 53850 Lappeenranta, Finland; soheil.aghajanian@lut.fi (S.A.); tuomas.koiranen@lut.fi (T.K.); 3 Department of Computer Science and Engineering, Chalmers University of Technology, 41296 Göteborg, Sweden; yuchong@chalmers.se (Y.Z.); fjeld@chalmers.se (M.F.)

**Keywords:** electrical resistance tomography, visualization, crystallization process monitoring, process operation

## Abstract

In the current research work, electrical resistance tomography (ERT) was employed for monitoring and visualization of crystallization processes. A first-of-its-kind MATLAB-based interactive GUI application “ERT-Vis” is presented. Two case studies involving varied crystallization methods were undertaken. The experiments were designed and performed involving calcium carbonate reactive (precipitative) crystallization for the high conductivity solution-solute media, and the cooling crystallization of sucrose representing the lower conductivity solution–solute combination. The software successfully provided key insights regarding the process in both crystallization systems. It could detect and separate the solid concentration distributions in the low as well as high conductivity solutions using the visual analytics tools provided. The performance and utility of the software were studied using a software evaluation case study involving domain experts. Participant feedback indicated that ERT-Vis software helps by reconstructing images instantaneously, interactively visualizing, and evaluating the output of the crystallization process monitoring data.

## 1. Introduction

Crystallization is a key process extensively used in many pharmaceutical product manufacturing and chemical applications. The process monitoring and control of crystallization techniques in industry have been subjects of study for many years [1]. There are primarily four types of crystallization methods: cooling crystallization, evaporative crystallization, anti-solvent based crystallization, and reactive crystallization [2]. These different types of crystallization processes involve various physical principles in the purification and separation processes.

The growing industrial demand for reactive type crystallization (also known as precipitation) is primarily due to the increasing demand for process intensification, yield efficiency enhancements, and lower energy consumption requirements [3]. In reactive crystallization processes, the main driving force is a fast chemical reaction [4]. Due to chemical reactions, crystal nucleation and growth phenomena are very fast, requiring an immediate response from process monitoring systems [3]. In reactive crystallization, instantaneous reactions cause differences in the density gradient within the reactor and the resultant solid product causes local variations in conductivity distribution within the suspension.

Due to the underlying physical and chemical differences, and certain operational challenges, various instrumentation has been employed to monitor the course of crystallization processes [5,6]. Collectively, crystallization monitoring and particle characterization techniques are known as process analytical technology (PAT) [7,8]. A range of PAT-based monitoring and feedback-control approaches have been utilized to investigate laboratory and industrial scale crystallization processes [9,10]. Widely used PAT instruments include particle vision microscopy (PVM) with image and data processing algorithms [11,12], visible light spectroscopy [13], impedance spectroscopy [14,15], focused beam reflectance measurement (FBRM) [16,17] and Raman spectroscopy [18]. These PAT tools significantly improve the design of unit operations and are valuable for crystal morphology assessments, including chord length and crystal count measurement.

Crystallization measurement tools such as IS and FBRM can obtain useful one-dimensional (1-D) and point-based information from the process. Alternatively, tomographic sensors have the potential to provide multi-dimensional data using reconstructed images [19]. Fast tomographic imaging techniques have important applications in industrial process control [19]. Tomographic techniques using hard field imaging and soft field imaging have been studied for crystallization monitoring. Hard field tomographic imaging uses ionizing radiation, and soft field tomographic imaging involves non-ionizing radiation. Hard field imaging techniques such as X-ray microtomography, and X-ray diffraction tomography have also been utilized to study microparticulates and crystallization processes [20,21]. Soft field tomographic imaging techniques, such as ultrasound computed tomography (USCT) [22] and electrical capacitance tomography ECT [23], have been widely described for monitoring crystallization progress. In batch and semi-batch chemical processes involving stirred tank reactors, it is important that the reconstructed images provide quantitative and accurate visualizations of the process. Continuous measurement can improve the implementation of process control.

As a complementary technique, tomography has the potential to provide useful information regarding qualitative determination of the spatial distribution of solid particles in a reactor, and to be utilized as a real-time fault detection and monitoring tool.

Electrical resistance tomography (ERT) is an inexpensive, fast, and non-destructive method for evaluating crystallization process. Because ERT depends on the inverse imaging methodology for reconstruction, there is a possibility of difference in sensitivity within the batch reactor at different distances from the sensor. The final results determining accurate yield estimation and solid concentration distributions within the region of interest can be affected by variation in the parameters for image reconstruction or image segmentation. The quantitative evaluation of the crystallization progress and the factors affecting crystallization monitoring using ERT have been discussed extensively [24,25]. It was shown that multiple factors such as sensor size, reconstruction technique, conductivity of the solution under evaluation, elements in the Finite Element Model (FEM) mesh, and the image processing method utilized all affected the quantitative evaluation of the non-conductive regions. 

It is a repetitive and time-consuming process to choose various parameters for different conductivity media for ERT image reconstruction. It is important that the acquired results be repeatable and that the evaluation protocol can be implemented to solutions with different conductivity profiles. The conductivity profile of the crystallization process in turn depends on the chemical and physical properties of the reactions involved. For instance, a reactive crystallization typically has fast kinetics in a high conductivity solution, while the sucrose crystallization by comparison has slow kinetics in a low conductivity medium. Hence, it is necessary to implement an ERT sensor which can provide resolution within the conductivity range of the given crystallization process [26].

Finally, the importance of advanced visualization for fast industrial tomographic processes to analyze objects under consideration has increased to a significant extent [27,28]. Therefore, there is a need to develop comprehensive and easy-to-use graphical-user-interface-based software with functionality of data acquisition, image reconstruction, image segmentation, and solid concentration distribution visualization.

This work aimed to demonstrate the versatility and capability of ERT for visualizing crystallization processes with different conductivity profiles and physical principles. In this work, an original software application called ERT-Vis was developed using MATLAB (Release R2021) with a novel approach for crystallization process monitoring and ERT data visualization. The applicability of the software for crystallization process monitoring was successfully tested using two experimental case studies, involving reactive crystallization and sucrose cooling crystallization, and a software evaluation case study involving domain experts.

The software development and experimental work were performed within the framework of the European Union Horizon 2020 TOMOCON project (smart tomographic sensors for advanced industrial process control) [19,29]. The primary focus of the project was to study fast tomographic methods and create a multi-sensor network to monitor, visualize and implement control for batch crystallization processes [29]. The present work is a collaborative effort from three partner universities within the TOMOCON project. The Lodz University of Technology (Poland) developed the ERT-Vis software and demonstrated the case study involving sucrose crystallization, Lappeenranta University of Technology (Finland) carried out a case study involving the precipitation of calcium carbonate, and Chalmers University of Technology (Sweden) performed the evaluation of the ERT-Vis software in a case study involving domain experts.

## 2. ERT System and Methods

### 2.1. Process Engineering Workflow Using ERT

**Figure 1:**
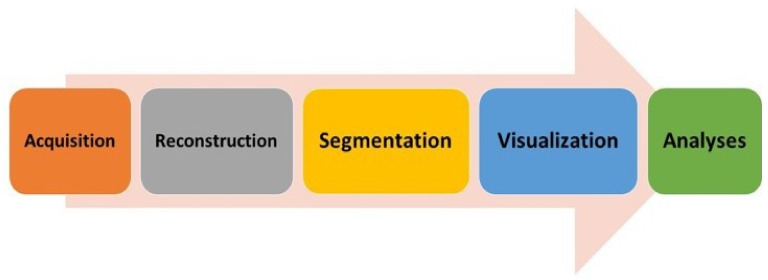
Process engineering ERT workflow (left to right): data acquisition, image reconstruction, image segmentation, 2D/3D visualization, and process analyses for control and monitoring.

The process engineering workflow for a PAT method based on tomographic image analysis can broadly be divided into five major segments, as shown in Figure 1. The factors determining quantitative accuracy using ERT evaluation fall into these five segments. The workflow starts with acquisition, where the selection of the number of electrodes, materials used for making an electrode, sensor shape, and frame rate of acquisition is determined. Any process analysis using ERT must include these steps in order to assess progress quantitatively. It is important for a process engineer that the control of these areas is provided separately so that the calibration and quantification can be performed systematically.

### 2.2. General ERT System

The ERT systems consists of a complex set of sensors and data acquisition technology, and employ inverse imaging techniques to generate images and information from the acquired voltage or current levels. The systems are primarily based on Ohms law stating that the material poses resistance to the path of electric current. There are two ways in which data acquisition takes place: voltage induced and current measured (VI), and current induced voltage measured (IV). 

**Figure 2:**
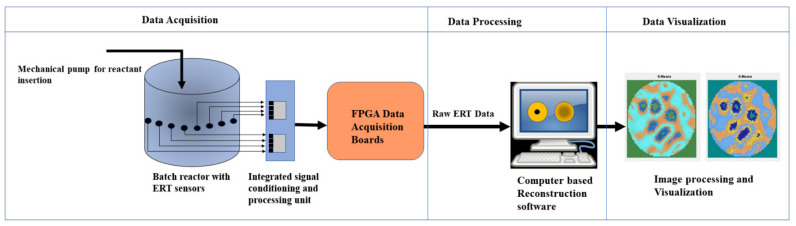
A schematic ERT system setup for reactive crystallization with data acquisition, data processing, and data visualization sections.

Figure 2 depicts the ERT setup schematic around a reactor tank. This setup can be categorized into three modular components: data acquisition, data processing, and data visualization. The data acquisition component consists of ERT electrodes placed on the circumferential periphery of the reactor. The sensor array consists of 16 steel electrodes. These electrodes operate as emitters (source) as well as receivers (sink) for the electrical signals. The cables carry the electrical signals via field programmable grid array (FPGA) boards. The FPGA boards remove any electronic noise received, by implementing filtering algorithms as a pre-processing strategy. The data processing such as image reconstruction and image processing techniques can be applied to the incoming data in real-time or in offline mode. The resulting images are visualized and analyzed, and the signals are sent back to implement the control strategy in the reactor.

### 2.3. Related Works for ERT Software Development

Soft field tomography involves reconstruction based on inverse imaging. It includes various non-ionizing tomographic methods such as ERT, ECT, USCT, microwave tomography and optical coherence tomography (OCT). Various software solutions from research labs and industry have been developed for analysis of ERT or ECT data. A short comparison of ERT software based on the available modules for process analysis is shown in Table 1. PyEIT is open access software for ERT reconstructions [30]; it is based on the Python programming language and offers simple 2D and 3D meshing. GREIT is software based on EIDORS for the monitoring of the thoracic region [31]. It is worth noting that GREIT is capable of reconstructing non-regular shapes, which could be beneficial in the vertical monitoring of batch reactors or irregular shapes. The ITS Reconstruction tool suite is an ERT software development for use with ITS industrial-grade ERT instruments [32]. It offers multiple reconstruction algorithms for the comparison of process data. Real-time 3D ECT was developed to obtain fast reconstructions of ECT images [33], using efficient GPU and CPU memory allocations for fast rendering of the 3D volumetric images obtained. TomoKIS studio is a software application developed at Lodz University of Technology [34]. TomoKIS can be connected to multiple ERT and ECT instruments in the process tomography laboratory, so that fast and efficient rendering of 2D and 3D images can be visualized at real time. It also supports multiple reconstruction algorithms for ECT data. EIDORS is an open source extensible software package for ERT and OCT reconstruction [35,36,37,38].

## 3. Experimental Setup and Crystallization Process Description

The Rocsole ERT device (supplied by Rocsole Ltd., Kuopio, Finland) was utilized during the experimental works. The ERT device was a voltage induced and current measured type. It was manufactured by Rocsole Ltd., Finland. A specific type of FPGA-based signal acquisition and transmission sensor unit was used to evaluate the signals in the low conductivity solutions (supplied by Rocsole Technology Centre, Rocsole Ltd., Kuopio, Finland). Sensors were mounted around the perimeter of the reactor with a diameter of 200 mm to monitor the calcium carbonate reactive crystallization.

Two case studies were performed to test the ERT-Vis software utility. The first case involved the CaCO_3_ reactive precipitation crystallization experiment, which used a higher conductivity medium. The second case study was the cooling crystallization process using the super-saturated sucrose solution with a relatively lower conductivity medium. Table 2 shows the difference between certain parameters for the investigated crystallization methods. The experimental setup is explained in Section 3.1 and Section 3.2.

### 3.1. Process Description of CaCO_3_ Reactive Crystallization

The CaCO_3_ reactive crystallization occurs by the addition of aqueous ${\mathrm{CO}}_{3}^{2-}$ into a stirred tank reactor containing a known concentration of calcium ions (calcium chloride was used as the calcium ion source). The governing chemical reaction is as follows:

(1) $${\mathrm{CO}}_{3\left(\mathrm{aq}\right)}^{2-}+{\mathrm{Ca}}_{\left(\mathrm{aq}\right)}^{2+}\to {\mathrm{CaCO}}_{3_{\left(s\right)}}↓$$

**Figure 3:**
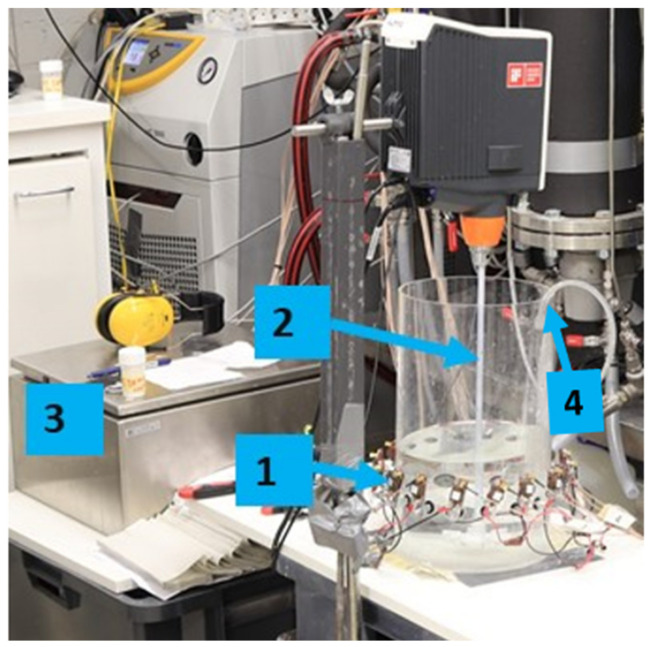
(1) Reactor with the ERT sensor unit mounted; (2) Rushton plastic turbine; (3) ERT device for data acquisition and process monitoring; (4) feed pipe for the reagent addition.

The rapid liquid-phase chemical reaction results in the formation of a non-conductive solid phase in the reactor. The initial solution volume inside the reactor was 3 L, as shown in Figure 3. The ${\mathrm{CO}}_{3}^{2-}$ reagent addition volume was 0.4 L (feed pipe diameter was 2 mm). For all the investigated cases, ${\mathrm{CaCl}}_{2}$ (purity > 98%, Merck, Darmstadt, Germany) concentration was 1.6 ${g\;L}^{-1}$, mixing speed was 100 RPM (tip speed of 0.37 ${m\;s}^{-1}$) and the feed addition rate was 40 mL${\;\min}^{-1}$. The aqueous ${\mathrm{CO}}_{3}^{2-}$ was prepared by injecting ${\mathrm{CO}}_{2}$ gas (purity > 99.9%) into sodium hydroxide (NaOH, Purity > 98%, Merck) solution—a detailed experimental procedure is provided in [39].

Experiments were performed at a temperature of (20 ± 2) °C. The reactive crystallization experiments and the associated ERT-based measurements were repeated at least three times to ensure the reliability of the results.

### 3.2. Process Description for Sucrose Crystallization

Sucrose (C_12_H_22_O_11_) crystallization using the cooling crystallization method involves the cooling of the saturated sucrose solution [40]. The coefficient of supersaturation k is expressed by the ratio

(2) $$k=\frac{\frac{\mathrm{Wsucrose}}{\mathrm{Wwater}}\mathrm{in}\;\mathrm{given}\;\mathrm{solution}\;\mathrm{at}\;T\;{}^{\circ}C}{\frac{\mathrm{Wsucrose}}{\mathrm{Wwater}}\mathrm{in}\;\mathrm{the}\;\mathrm{saturated}\;\mathrm{solution}\;\mathrm{at}\;T\;{}^{\circ}C}$$

where W_sucrose_ is weight of the sucrose in the solution, W_water_ is weight of the water in the solution, and T is the temperature of the solution. Experimental data on the solubility of sucrose in pure and impure solutions at various temperature has been widely reported in the literature [41,42].

**Figure 4:**
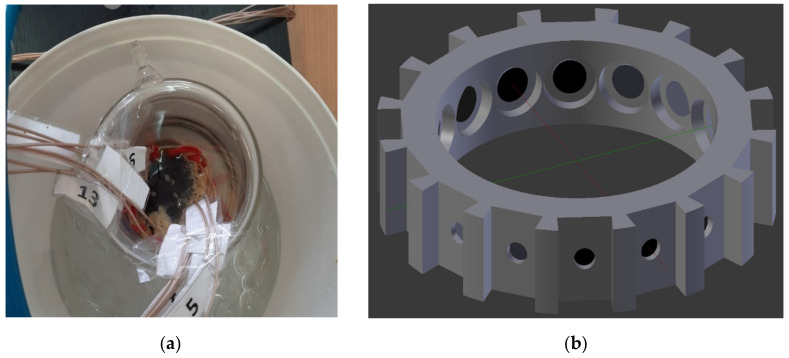
(**a**) Jacketed beaker with supersaturated sucrose solution inside the ice bath (coaxial cables are numbered from 1 to 16); (**b**) 3D printed sensor insert, to mount the ERT electrodes.

Percentage of mass of soluble sucrose up to 100 °C is given as in [41]

w_S_ = 64.447 + (0.08222 × T) + (1.66169 × 10^−3^ T^2^) − (1.558 × 10^−6^ T^3^) − (4.63 × 10^−8^ × T^4^), (3)

where w_S_ is the percentage in the mass of soluble sucrose and temperature in °C is given by T. Using the jacketed glass beaker as shown in the Figure 4a, a design was proposed to perform the cooling of the saturated sucrose solution. As the name suggests, the jacketed beaker has a temperature-maintaining glass jacket around the reactor. The outer height and the outer diameter of the beaker measured 195 mm and 120 mm, respectively. The inner height and the inner diameter of the beaker measured 175 mm and 95 mm, respectively. A challenge of using the glass reactor involved the difficulty of drilling holes for ERT sensor placement as used in the reactor made from polymer material. Hence, a novel design for the placement and insertion of the ERT sensor unit was 3D printed, as shown in Figure 4b. The sensor was placed within the beaker’s circumference. Black non-conducting paint was applied on the reverse to prevent leakage of current. The MCX coaxial connectors were connected to the Rocsole device. The coaxial cables were soldered to the sensor and a rubber insulation was provided to avoid any contact with the supersaturated solution, which would result in noise in the acquired signal.

The 3D sensor insert was designed using Blender version 2.79b software. It was 3D-printed using Ultimaker version 3 Extended software with an accuracy of 1 mm, with the help of Cura 4.6 software. The sensor insert was printed using acrylonitrile butadiene styrene (ABS) material. The jacketed beaker was filled with water at 0 °C and placed in an ice bath to maintain constant temperature. Saturated sucrose solution weighing 400 g was prepared from Polski Cukier sugar crystals and tap water. The solution was heated to 90 °C and poured inside the beaker, and measurements were taken at reducing temperatures of 90 °C, 45 °C, 40 °C and 35 °C.

## 4. Development of the Software ERT-Vis

Electrical Resistance Tomography (ERT) can provide 2D/3D images supporting analytic tasks for chemical process analysis. Effective use of such images is critically reliant on the choice of reconstruction parameters and the flexibility to change them quickly. We systematically studied such parameters for analyzing non-conductive materials in the low conducting media [25]. For cylindrical chemical batch crystallization reactors, a conventional parameterization approach relies on testing and comparing the number of iterations, finite element model mesh structure, hyperparameter values, and tolerances, using simulations and phantoms. We conjecture that the interactive parameterization of segmentation methods and morphological image processing will be critical for evaluating the spatial accuracy of reconstructions in low conductivity environments. A visual analytics-based software ERT-Vis is presented, for visualizing reconstructed ERT images and applying run-time image processing techniques. This software consists of four modules for ERT data analyses: acquisition, reconstruction, segmentation, and visualization. A software evaluation case study involving domain experts was also conducted.

There has been an imperative need to develop a versatile software to help address the unique requirements of the process engineer. Such software must be able to acquire data from ERT, to reconstruct an image according to the flexible parameters chosen by the process engineer, to perform the image processing tasks and provide flexibility to visualize data in the requisite format, depending on the type of crystallization experiment performed. The human–computer interaction was an essential part of the experiment, as the requirements of the process engineer vary for different crystallization methods. The developed software was tested with the involvement of domain experts from the field of tomography. Based on the common feedback from the domain experts, a special module for generating videos was added.

### 4.1. Development of the Application Modules and GUI

ERT-Vis is a MATLAB-based software application created using the MATLAB app-designer toolbox. The GUI tool ‘UIAxes’ was extensively used to display plots, panels, and reconstructed and segmented images. A general selection header strip can be seen across all the software modules for interactive selection, activation of modules, and navigating through image frames either sequentially or to a specified frame. The START ERT-Vis push button must be pressed to initialize the ERT-Vis software from the general selection header strip before beginning to use the application to initialize the libraries.

Four modules were implemented in the prototype software, in line with the data workflow in the ERT data acquisition and analysis system. These modules differed in their functions and were assimilated into separate tabs for better accessibility. In the current version of the ERT-Vis software, a researcher option has been added for enabling and disabling the four modules by pressing the activate push buttons in the general selection header strip. This saves time by reducing the number of computations required to update the displayed plots and images. With the “Activate Acquisition” button, the acquisition module tab is rendered functional. Using the “Activate Reconstruction” button, the reconstruction capabilities of the ERT-Vis software in the reconstruction tab are switched on. With the “Activate Segmentation” button, the image-processing capabilities from the MATLAB image processing toolbox are utilized to segment and process the data. With the “Activate Visualization” button, users have the capability to visualize the data in various available colormaps of the extracted individual RGB channels of the image, and can implement binarization on the extracted images by grey-level thresholding or flood-fill segmentation.

The general selection header also consists of the reconstruction status LED indicator. This LED blinks red prior to the execution of the reconstruction algorithms and turns green when the reconstruction is completed. The default status of the reconstruction LED is yellow. Using the “Frame Select” slider, the current frame under observation can be moved to the desired location. The range of the “Frame Select” slider value is set from 0 to 900 and can easily be changed via the MATLAB App designer. Alternatively, researchers can automate the process, using the designated variable to track the number of frames in the uploaded data. The spinner “Frame Step” accepts the numerical input within the range of “Frame Select” slider values, and reconstructs the user-defined frame. This feature is convenient for observing minor changes that occur within microseconds of the process with fast kinetics, acquired using a high frame-rate ERT acquisition device one frame step at a time.

### 4.2. Module 1: Data Acquisition

**Figure 5:**
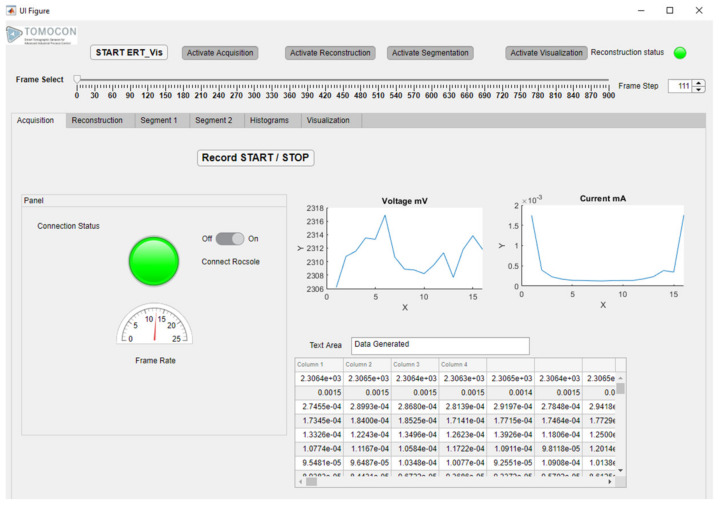
ERT-Vis: Data acquisition module tab at initial condition.

In this tab, data can be acquired using the ERT device online over a Wi-Fi connection or via LAN connection. The “Data Acquisition” tab can be seen in Figure 5. The “Data Acquisition” module of the ERT-Vis was tested as an independent module using the Rocsole device at Lodz University of Technology. At the LUT University the ERT device was connected with LAN connectivity. At the Lodz University of Technology the ERT data was obtained by forwarding it via the in-house TomoKIS Studio software [34], which was physically connected to the Rocsole device through an internet router. Rocsole Ltd. provided DLLs for enabling the connection of the ERT device to the TomoKIS studio software. The case study evaluations for cooling crystallization using the ERT-Vis were carried out using the recorded data.

Using the “on–off” toggle switch the user can connect to the ERT device over Wi-Fi. The status of the ERT device connection is indicated using the colored LED “Connection Status”; disconnection is indicated using red, and the LED status indicator turns green if the PORT status is open and the device is connected. The default status before the first reconstruction is yellow. The currents acquired and the voltages of the frame are visualized in the “Currents” and “Voltages” plot. The numerical streaming data can be seen in the table columns below the plots in the MATLAB table. The streaming data is saved into the text format using the “Record START/STOP” button.

### 4.3. Module 2: Reconstruction

In this module, the main task of the reconstructions from the ERT data are achieved. The images obtained after the ERT reconstruction depend on various factors such as hyperparameter values, the number of iterations, the FEM mesh model structure, the number of sensors, and the number of pixels in the resultant image. Within this module, the user has flexibility to choose the reconstruction method and to make fine adjustments to achieve better results and to visualize results immediately and interactively.

**Figure 6:**
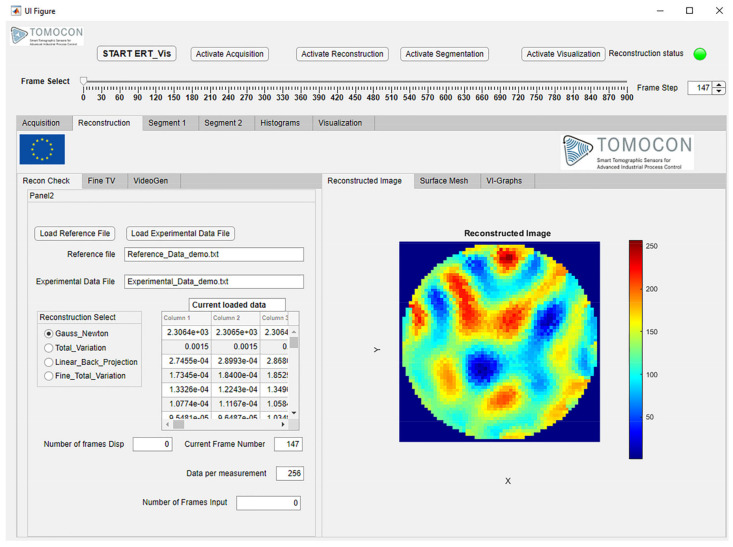
ERT-Vis Reconstruction module tab.

The “Reconstruction” tab is shown in Figure 6. In this version of ERT-Vis, three reconstruction algorithms have been implemented: the Gauss-Newton (GN) algorithm, the Total-Variation (TV) algorithm and the Linear Back Projection (LBP) algorithm. These algorithms have been implemented using EIDORS open-source software. EIDORS version 3.10 is central to the reconstruction module for ERT-Vis [43]. EIDORS is an open-source MATLAB toolkit for electrical resistance tomography [44]. It approaches nonlinear or ill-posed problems in electrical resistance or electrical capacitance tomography using a finite element model (FEM) for forward calculations. A regularized nonlinear solver is implemented to obtain a unique and stable inverse solution. This includes a derivation of the formula for the Jacobian matrix or the sensitivity matrix, based on the complete electrode model.

**Figure 7:**
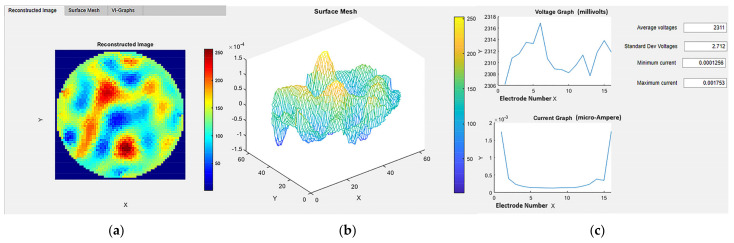
Reconstruction module: Panel for advanced visualization tabs (**a**) 2D reconstruction; (**b**) 3D surface mesh visualization; (**c**) graphical and numerical observations.

The “Reconstruction” tab is vertically divided into two sections. This tab is activated after pressing the “Activate Reconstruction” state button. In the “Recon Check” tab of the reconstruction module on the left, the reference data and the experimental data can be imported using the push buttons “Load Reference File” and “Load Experimental Data File”, respectively. The file names of the imported experiment are displayed and verified in the Edit Text Field boxes “Reference File” and “Experimental Data File”, respectively. The reconstruction algorithm can be selected from the four options currently provided in the button group “Reconstruction Select”. The change in the selection of the “Reconstruction Select” button group results in the generation of a new image in the “Reconstructed Image” tab on the right-hand side. The numerical edit text fields “Current Frame Number”, “Data per Measurement”, and “Number of Frames” display the current frame monitored, data points in the single measured frame, and the total number of frames in the current experimental dataset, respectively. In the right-hand side section, as shown in Figure 7, the 2D reconstructed image is observed in the “Reconstructed Image” tab; this provides us with a 2D visualization. In the “Surface Mesh tab”, a 3D surface mesh provides a 3D visualization. Information regarding the induced voltage stability using the average and standard deviation of the voltages in the frame can be observed numerically in the “VI-Graphs” tab. Information regarding minimum and maximum current in the frame is also observed in this tab, to check the sensor capabilities for detecting the currents in the solution provided.

**Figure 8:**
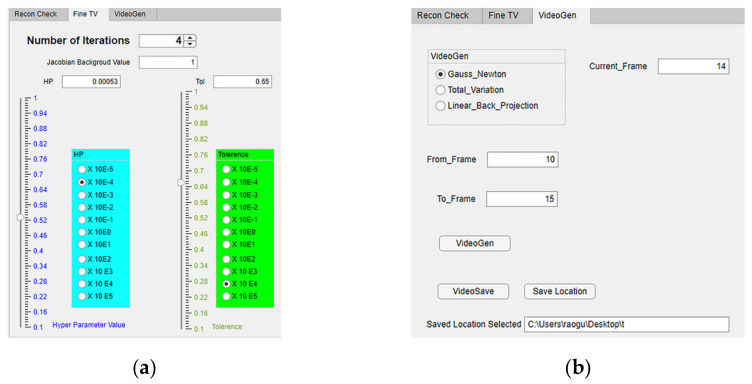
(**a**) Reconstruction module: Total Variation algorithm tab (selecting reconstruction parameter value progression from coarse to fine/detailed); (**b**) VideoGen function in ERT-Vis.

Additionally, the reconstruction algorithm “Total Variation” can be controlled from the “Fine TV” tab on the left axes as shown in Figure 8a. Here the number of iterations varies according to the spinner “Number of Iterations”. The Jacobian background value can be edited using the numerical edit field “Jacobian Background Value”. The hyper-parameter value and tolerance can also be varied from 1 × 10^−5^ to 1 × 10^5^ with the help of the separate sliders and the multiplication factor selected from the button group. They are color-coded blue and green for easy access. The values set are visible in the numerical edit field boxes “HP” and “Tol”, respectively.

To observe precipitation as a frame-by-frame video, two new functionalities were added to the ERT-Vis software in the reconstruction tab, as shown in Figure 8b. These are called “VideoGen” and “VideoSave”. Using this functionality, a user can generate a video to observe the progress of the reaction from the saved data, and can save the video files. This helps fast analysis of raw data using different reconstruction techniques and application of various image-processing techniques. The user must input the range of frames for observation, into the “From_Frame” and “To_Frame” numerical input boxes, and provide the location after pressing the “Save Location” button, in case the video is required for further analysis.

### 4.4. Module 3: Segmentation

**Figure 9:**
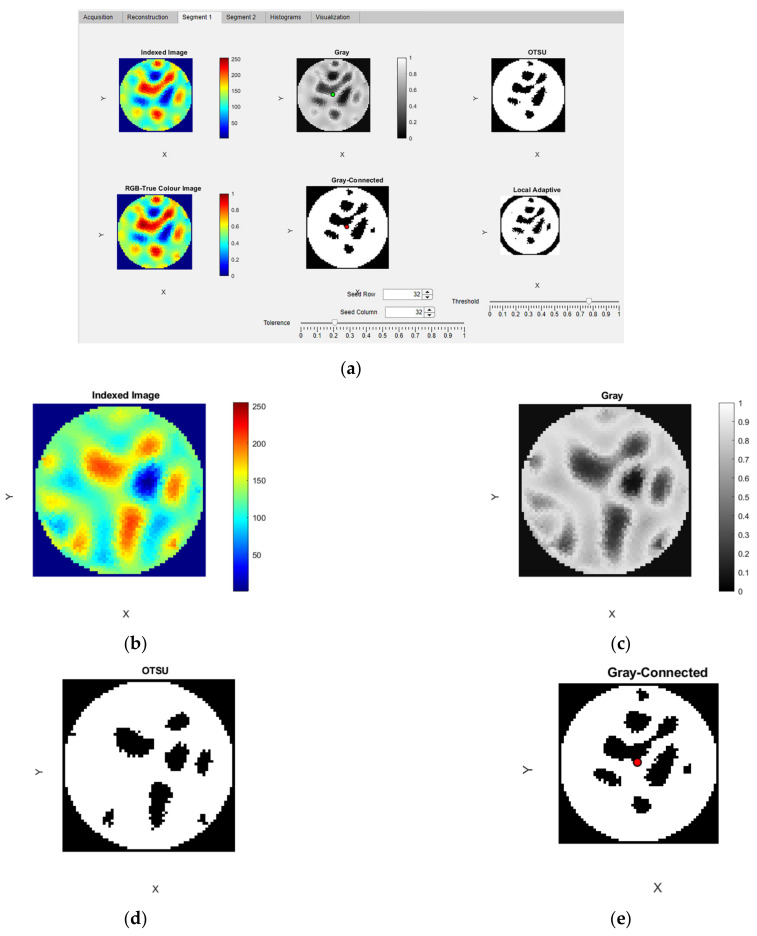
Segmentation module: (**a**) Segment 1 TAB; (**b**) indexed image; (**c**) gray image; (**d**) OTSU-segmented; (**e**) gray-connected segmented image with seed location.

In this module, there are two tabs for segmenting the reconstructed ERT image with crystal regions. In the “Segment 1” tab there are six panels, as shown in Figure 9. The output of the EIDORS software provides an indexed image which is mapped onto a ’jet’ colormap and displayed in the “Indexed Image” panel. This indexed image is converted to an RGB true color image using the function mat2im() [45]. The converted true-color image is visualized in the “RGB-True Color Image” panel. The indexed image is converted into a gray image using the MATLAB inbuilt function rgb2gray(). This resulting image is displayed in the “Gray” panel. OTSU segmentation is applied to this gray image using the MATLAB function otsuthresh() after evaluating the histogram using the function imhist. The resulting image is shown in the “OTSU” panel. The “Gray-connected” panel, displays the result from the flood-fill image segmentation performed using the function grayconnected() MATLAB function. This is an interactive segmentation method where the user provides interactive input. Three inputs are required for this segmentation to operate: The row number, the column number and the tolerance. The row and column input for the initial seed point are applied using the spinners “Seed Row” and “Seed Column” within the range of 0 to 64. The tolerance for the range of gray levels can be controlled within the range 0 to 1 using the slider “Tolerance” below the axis. In the “Local Adaptive” panel, the results from the MATLAB image segmentation function adaptthresh() are visualized. The threshold value for the binarization is controlled using the slider “Threshold” below the axis. In Figure 9a, the Segment 1 tab can be observed with six image displays showing results of various image processing algorithms. Figure 9b–e shows the resultant reconstructed images application of image processing algorithms.

**Figure 10:**
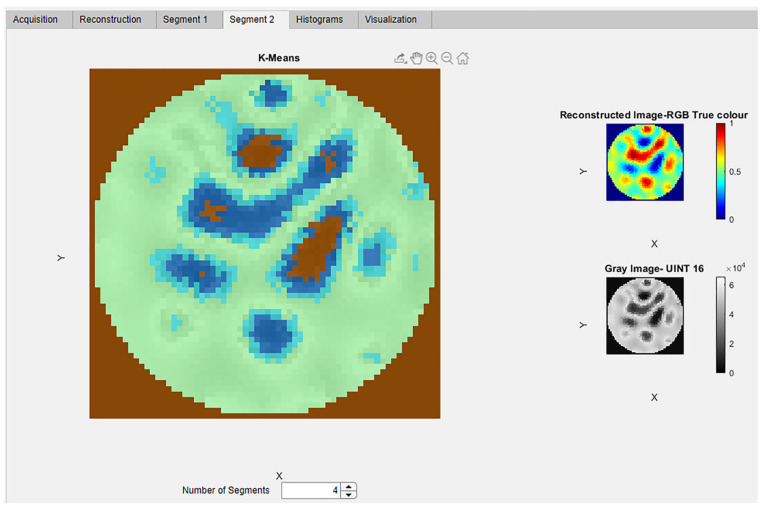
Segmentation module: Advanced segmentation tabs for observing the K-means clusters for the crystal regions. The reconstructed RGB image and gray image is also displayed in panel.

In the “Segment 2” tab of the segmentation module an advanced segmentation method of K-means clustering is provided. The k-means clustering in MATLAB is implemented using the function imsegkmeans. This is an advanced segmentation technique which segments image data using unsupervised learning. The user has the ability to provide the number of segments as an input. This tab consists of three display panels, as shown in Figure 10.

The ERT reconstructed image and 16-bit unit gray image can be visualized in the respective panels. The 16-bit gray image is obtained using the MATLAB function im2uint16. The Uint16 image is used to implement the K-means clustering. The spinner “Number of Segments” provides the input values for classifying the image into the various clusters. This provides the flexibility to classify and extract the cluster region of interest for further analysis and study. Different clusters are automatically color-coded for better visualization.

### 4.5. Module 4: Visualization

**Figure 11:**
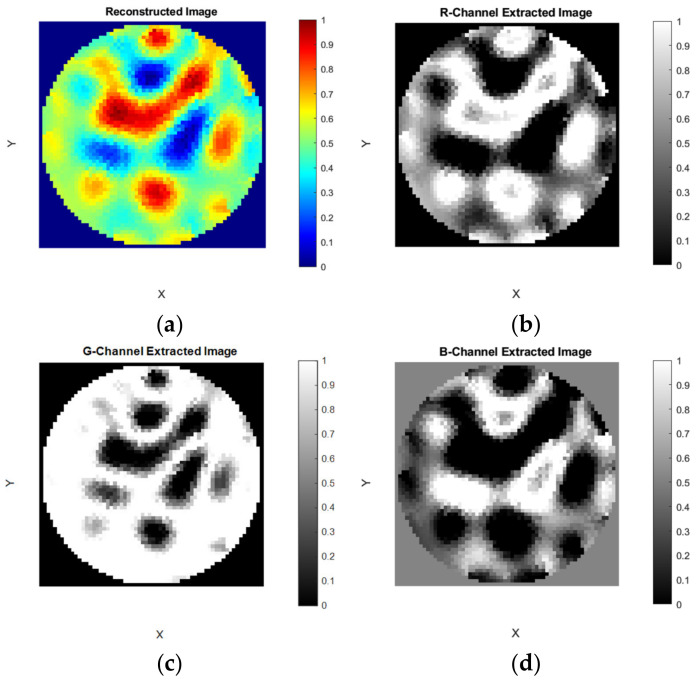
Visualization module: Color channel extraction and visualization subfigure (**a**) reconstructed image; (**b**) R-Channel image; (**c**) G-Channel image; (**d**) B-Channel image.

In the “Visualization” tab group there are seven sub-tabs. The first four tabs “RGB”, “R-Channel”, “G- Channel”, and “B-Channel” are visualization tabs. The next three tabs “R-Channel-binarize”, “G-Channel-binarize, and “B- Channel-binarize” are for advanced interactive visualization and segmentation of the extracted image color channels. In the tab “RGB”, the reconstructed image and the images extracted from the color channels are shown in four axes; “Reconstructed Image”, “R-Channel Extracted Image”, “G-Channel Extracted Image”, and “B-Channel Extracted Image”, as shown in Figure 11.

**Figure 12:**
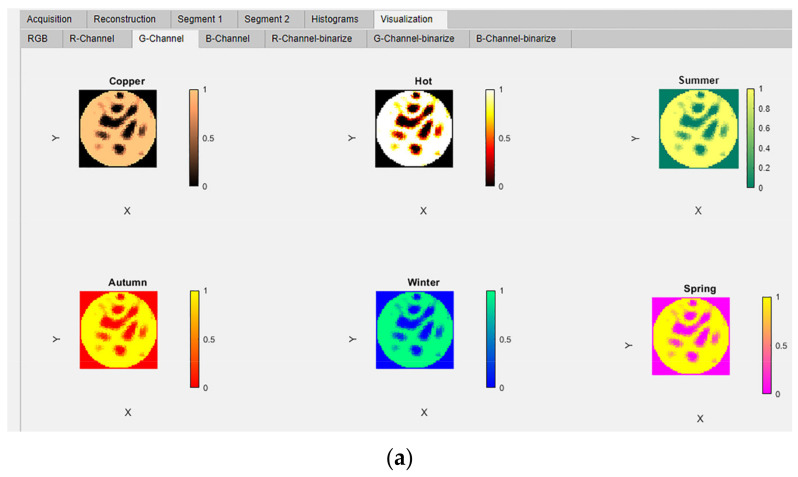

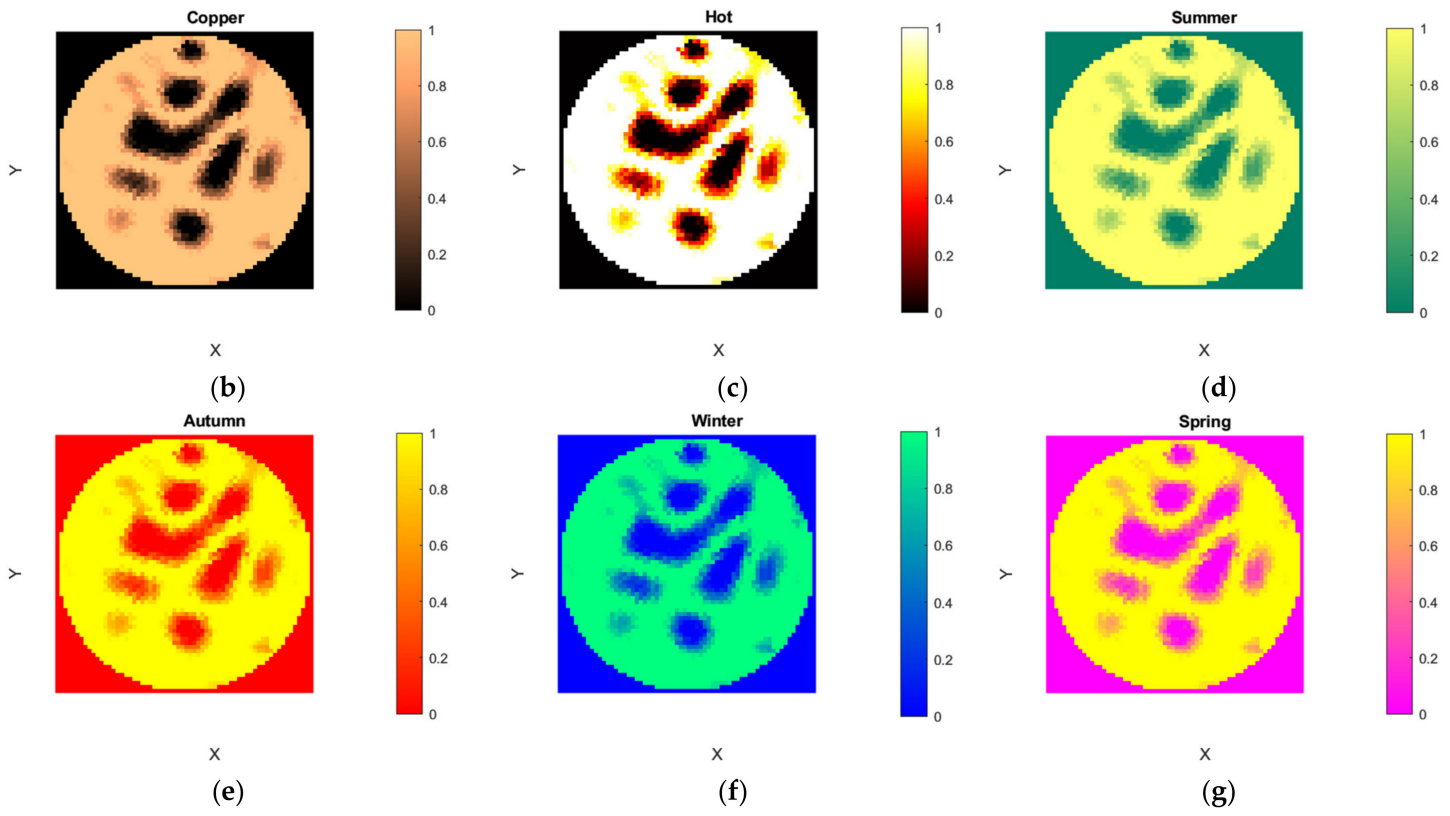
Visualization module: Simultaneous colormap observations subfigure (**a**) G-Channel observed with various colormaps, colormaps implemented: (**b**) copper; (**c**) hot; (**d**) summer; (**e**) autumn; (**f**) winter; (**g**) spring.

The ERT-reconstructed RGB true-color images were extracted into the three separate images and displayed in these panels using the MATLAB function imsplit. The extracted channels have been mapped to six different MATLAB colormaps; copper, hot, summer, autumn, winter, and spring [27]. These six colormap images are simultaneously displayed in six panels in the “R-Channel”, G-Channel, and “B- Channel” tabs. The titles of panels correspond to the names of the colormaps: “copper”, “hot”, “summer”, “autumn”, “winter”, and “spring”, as shown in Figure 12a–g.

**Figure 13:**
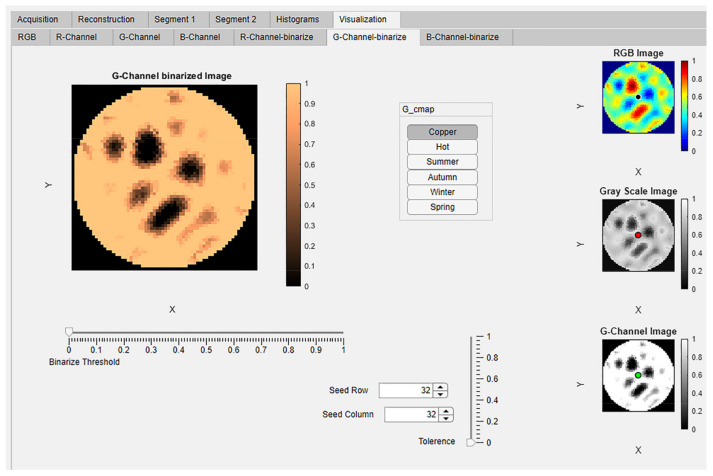
Visualization module: Advanced interactive visualization and segmentation tab.

The advanced interactive visualization and segmentation tabs have been designed for every extracted color channel of the image. This is visualized in the tab “G-Channel-binarize,” as shown in Figure 13. It contains four panels. “RGB Image”, “Gray Scale Image”, and “G-Channel Image” can be seen on the right vertical strip. The user can interactively select the colormap from the button group “G-cmap” to view the extracted color channel. Using the slider value from “Binarize threshold” as a threshold, the images are binarized using the MATLAB function imbinarize(). Interactive segmentation of the extracted color channel operates using the spinners “Seed Row” and “Seed Column” along with the slider “Tolerance” as input to the MATLAB function grayconnected(). The seed pointer location could be seen in blue color within RGB image, in red color within gray scale image, and in green color within the G-Channel image as shown in Figure 13.

## 5. Results

### 5.1. Software Evaluation Case Study

#### 5.1.1. Case Study

We demonstrated ERT-Vis with a case study involving four domain experts performing several tasks to evaluate the effectiveness of our application. The four participants are denoted as P1, P2, P3, and P4 respectively, and their individual domain backgrounds are elaborated below. The case study was successfully organized across various countries with the involvement of domain experts. Online co-ordination was achieved using MS-Teams software from Microsoft. One issue arising during the study was the limitations of the software on Mac computers. To overcome this, the domain experts were given remote access to the author’s laptop to conduct the tasks.

P1: PhD student who has been working with ERT for three years.
P2: Associate professor with over 15 years of experience in ERT technology.
P3: Professor with more than 20 years of experience in tomography.
P4: PhD student with almost three years of hands-on tomographic experience.

The case study comprised three parts: a preparation meeting, separate implementation with real-time feedback from each participant, and a post-feedback session. To start, each participant attended an initial session online and consented to be recorded over the whole process. In the preparation meeting, we clarified the relevant issues and then demonstrated a tutorial of ERT-Vis. Next, every participant was assigned a time slot and requested to perform an ERT visual analytics task including seven microtasks as illustrated below. Each expert received the same task list, but they obtained distinct results since they were asked to select different images at the beginning (denoted with “X” in the illustration). After completion, the participants had another opportunity to provide extra post-feedback, after previously having given real-time comments.

#### 5.1.2. The ERT Visual Analytics Task

Task-1: Load the reference data, then load the experimental data.
Task-2: Choose the frame number X using the slider.
Task-3: Check various image reconstructions. Check the 2D images and 3D meshes-V-I numerical data in different tabs.
Task-4: Observe the segmentation results. Switch to any other segmentation methods.
Task-5: Observe the histograms of the images.
Task-6: Observe the separated R, G, and B channels of ERT images.
Task-7: Select and change the colormaps of the extracted R, G, and B channels.
Task-8 (optional): Conduct binarization using the threshold and visualization for the gray-connected seed row/column.

The comments from participants regarding the various tasks were recorded as shown in Table 3.

#### 5.1.3. Insights

Timesaving: The primary characteristic reported by the participants regarding ERT-Vis was immediacy. They noticed that there were no built-in iterative algorithms to make the application display the results after changing the arguments. Different from other applications, ERT-Vis adopts a simple algorithm selection–result display strategy, ensuring that users can simultaneously choose the desired method then obtain the corresponding result in a short time. Based on the quick response time throughout the applications, the efficacy and efficiency of tasks improved remarkably.

Descriptive: The participants referred to the descriptive information included in ERT-Vis. Most of them indicated that ERT-Vis offers parallel analysis of data acquisition, reconstruction, segmentation, and visualization, which is a significant breakthrough in comparison to other tomography-related visual analytic tools they had used previously. The specific enrichment of each module was appreciated, as there are multiple approaches supplied in every module. For example, the users had the capability to choose diverse reconstruction and segmentation methods when carrying out the hands-on analyses. The workflow was well designed to support comprehensive visual analysis for ERT-related decision-making. In particular, P1 noted that he was astounded by the ’seed segmentation’ part, as it enables the users to gain a deeper understanding of the domain problems.

User-friendly: Overall, ERT-Vis was deemed a user-friendly application by the participants. They reported that the design of the GUI is intuitive and comprehensible, and agreed that ERT-Vis was easy to use throughout the whole operation period. The conciseness and transparency of the interface gave them a clear overview of each module, enabling them easily to grasp the functionality to proceed with their work. In particular, P4 was especially satisfied with the layout of ERT-Vis showing several output images side by side in the same interface. He felt it was straightforward and convenient to compare the results under such settings. The capacity of the system to toggle different reconstruction methods, segmentation methods, and visualization categories was highlighted by every participant. 

Limitations: Certain limitations regarding ERT-Vis were pointed out by the domain experts. Common requests included the facility to generate videos from entire frames and the possibility of saving images. More specifically, P3 indicated that the VI-graph should be designed as a tunable panel, which would allow users to better interact with the visualization results. P4 requested inclusion of a timestamp over the reconstructed image tab for comparison with future imaging modalities. As the frame rates increase, microsecond display would inform the user of the status of crystallization within time differences of microseconds. Prior smoothness selection has not yet been included. The 3D reconstruction modules and algorithms have not yet been implemented and will be incorporated in future iterations.

### 5.2. Results for CaCO_3_ Precipitative Crystallization Using ERT-Vis

Initially, a metallic impeller was utilized in the experimental setup. The reconstructions with the metallic impeller included significant noise during the acquisition of ERT signals. Therefore, a plastic-fabricated Rushton impeller was utilized for agitation. Using the ERT-Vis software, detection and resolution of the noise issue in reconstructed images were swiftly resolved, which optimized the overall time required for experimentation. Quick analysis prior to the start of the experiments provided us with an added advantage in performing closed-loop control experiments [26].

**Figure 14:**
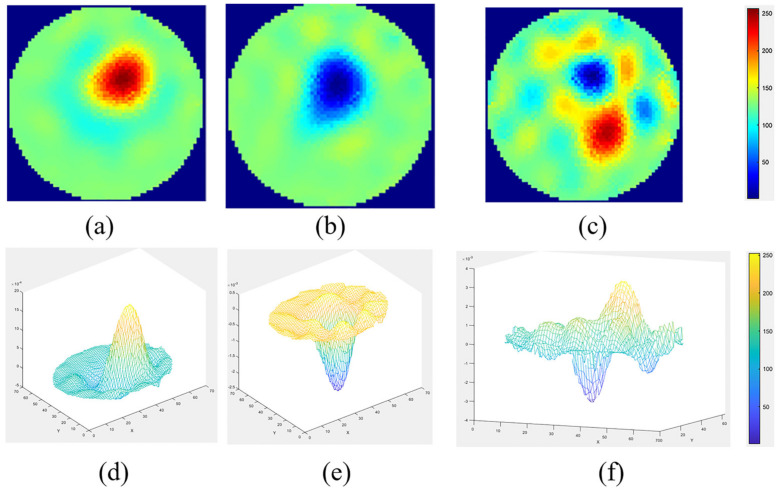
Reconstructed images and 3D surface mesh of (**a**,**d**) plastic stirrer, (**b**,**e**) metallic stirrer, and (**c**,**f**) plastic and metal stirrer together.

Figure 14 shows reconstructed images of plastic, metal, and plastic–metal together, along with the surface mesh. The images were reconstructed using the one-step Gauss-Newton reconstruction method. The difference between the metal and the plastic stirrer is observable. The metallic stirrer included noise in the ERT single electrode acquisition and in the reconstructions, hence the plastic stirrer was utilized. For the process engineer, this is important information to help avoid noise generated by the metallic stirrer.

**Figure 15:**
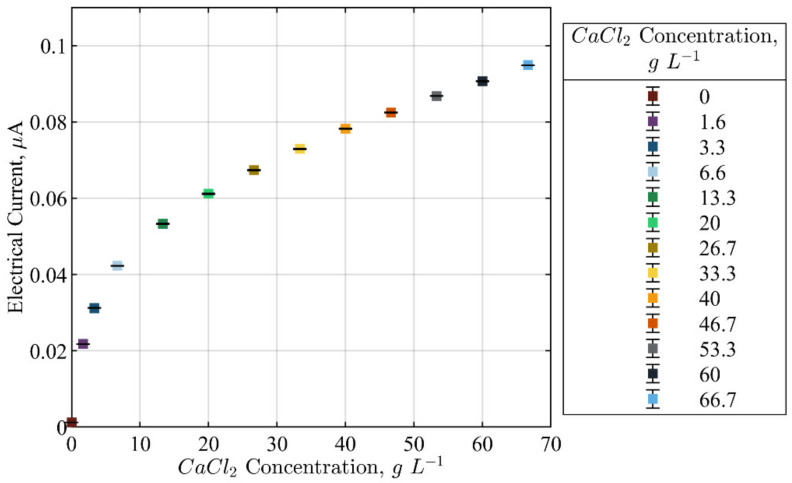
Average electrical current measurements evaluated for 100 frames for different concentrations of CaCl_2_ solutions with no stirrer motion.

The evaluation of the changes in electrical current due to the changes in the concentration of calcium chloride in the solution was tested. Figure 15 shows the changes in the average electrical current from 0 gL^−1^ to 66.7 gL^−1^. It was noted that the current changed from 0.02 µA to 0.1 µA. These tests proved that the FPGA signal conditioning units of the ERT device could resolve minor conductivity changes in highly conductive solutions involving calcium chloride.

**Figure 16:**
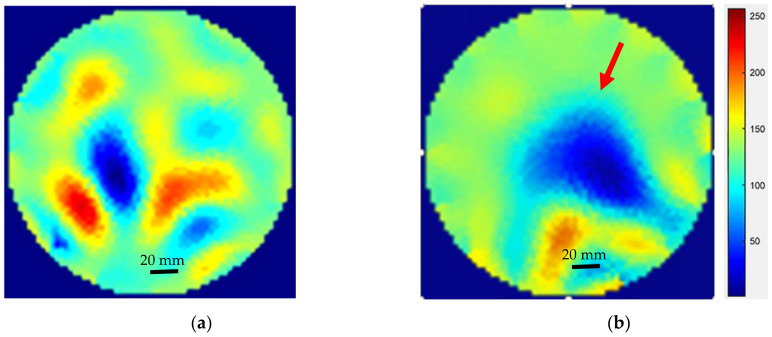
(**a**) Water inside reactor; (**b**) CaCO_3_ insertion detected using ERT (blue region indicated by arrow).

Further tests using ERT-Vis software were conducted to evaluate the detection of calcium carbonate CaCO_3_ inside the reactor. For this purpose, the VideoGen tool was used and the images were saved. The images prior to the addition of any crystal additives consisted of noise due to motion of water and the amplification of minor differences by the Gauss-Newton reconstruction algorithm, as shown in Figure 16a. Powdered calcium carbonate weighing 100 g (VWR, purity > 99%) was put inside the reactor and the images were reconstructed using the Gauss-Newton algorithm. The changes in the reactor were visible and the color of the reactor turned opaque. The solid microparticles of calcium carbonate appeared as a non-conducting region, as shown in Figure 16b.

**Figure 17:**
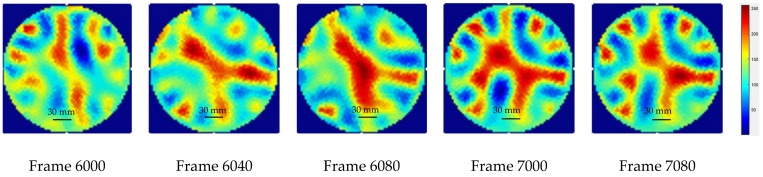
Observation of settling of CaCO_3_ in the suspension at various stages of the experiment. Red regions indicate the CaCO_3_ crystal regions.

Final tests were completed using the ERT-Vis software to detect the presence of calcium carbonate in the base solution of NaOH and calcium chloride, to detect the presence of calcium carbonate crystals. Figure 17 shows the ERT reconstructed images for the observation of the settling of CaCO_3_ within the reactor. The calcium carbonate CaCO_3_ particles can be observed in the red colored areas. As the time progresses, we can see the precipitation bolus move downwards in the reactor.

**Figure 18:**
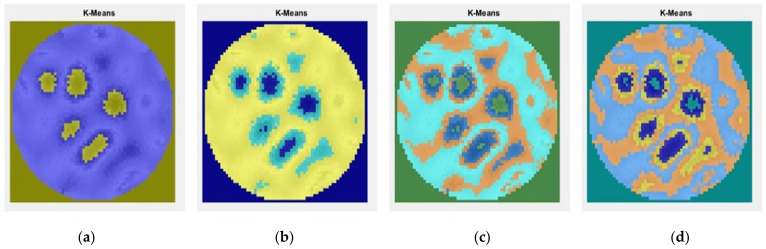
Segmentation module: Interactive observation of the K- means cluster regions (**a**) two clusters; (**b**) three clusters; (**c**) four clusters; (**d**) five clusters.

To determine the calcium carbonate presence in the solution using the unsupervised learning method, K-means clustering segmentation was implemented. Figure 18a–d shows the effects of changing the number of clusters in the image to two, three, four, and five clusters.

### 5.3. Results for Sucrose Crystallization Using ERT-Vis

**Figure 19:**
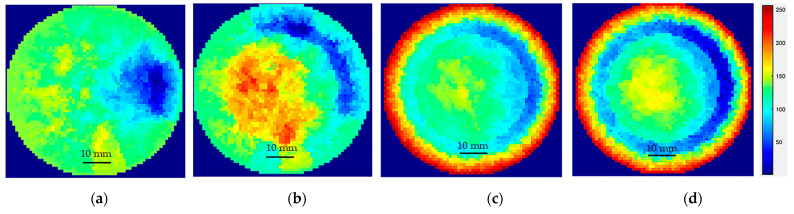
Blocking of the sensor due to sucrose crystallization over the ERT electrodes (**a**) 90 °C; (**b**) 45 °C; (**c**) 40 °C; (**d**) 35 °C.

The results for temperatures from 90 °C to 18 °C are presented in Figure 19a–d. It can be seen that at 90 °C, the measurements showed a certain discontinuous region inside the reactor. These regions indicate the onset of crystallization over the electrodes. At 45 °C, some low conductivity regions were visible, but the reconstructed images had significant noise. At 40 °C and 35 °C the sensors were completely blocked by crystal formation over the electrodes and no electrical signal could pass through.

## 6. Discussion

In this contribution, ERT-Vis has been presented as a novel interactive application designed to facilitate Electrical Resistance Tomography (ERT) data visualization and evaluation. ERT-Vis is an open-source MATLAB-based application software. The ERT-Vis software is versatile and extensible; it addresses a range of ERT process engineering and data visualization purposes. The primary contribution of ERT-Vis is that it enables rapid prototyping of different conductivity profiles, acquired using an ERT device. This is useful when searching for the most efficient reconstruction–segmentation–visualization workflow for a new liquid media or solid–liquid mixture.

The presented application case study involving domain experts proved to be useful in determining the utility of the application for crystallization process monitoring. We envision numerous possibilities using refined ERT-reconstructed image data for data processing and implementation of control models and machine learning models. ERT-Vis can help researchers in streamlining tasks at hand quickly and enable them to focus more on their analysis of the process data acquired. Based on responses from the case study, a tool was developed for obtaining a video file for the selected range of frames. We foresee implementing further EIDORS functions into ERT-Vis, as well as analyses based on unsupervised learning. Such functionality will be offered as a user-friendly GUI for process applications. We also foresee keeping the software open-source for further developments. This software has the potential to be further developed as a cloud-based service for industrial applications.

## Figures and Tables

**Table 1:** Comparison of ERT software based on the availability of different modules.

Software	Acquisition	Reconstruction	Segmentation	Visualization	Researcher’s Open Access
pyEIT	No	Yes	No	Limited	Yes
GREIT	No	Yes	No	Limited	Yes
ITS Reconstruction Tool-Suite	Yes	Yes	Limited	Limited	No
Real-time 3D ECT	Limited	Yes	No	No	No
TomoKIS Studio	Yes	Yes	No	Limited	No
EIDORS	No	Yes	No	Limited	Yes
ERT-Vis	Limited	Yes (EIDORS)	Yes	Yes	Yes

**Table 2:** Case studies and experimental configuration to demonstrate the application of ERT-Vis software in different crystallization processes.

Parameter	CaCO_3_ Reactive Crystallization	Sucrose Cooling Crystallization
Size of Reactor	200 mm diameter	63 mm internal diameter
Number of Electrodes	16	16
Type of Reactor	polypropylene	Glass jacketed
Acquisition Frame Rate	16 Hz	12 Hz
Reconstruction Algorithm	Gauss-Newton	Gauss-Newton
Total Time for Experiment	10 min	15–20 min
Type of Crystallization	Reactive crystallization	Cooling crystallization
Stirrer Speed	100 rpm	No stirrer
Input Induced Voltage	3 V	3 V
Range of Currents detected	0–0.1 µA	0.1–1.75 mA
Transducers Frequency	156 KHz	156 KHz

**Table 3:** Comments from various experts for the assigned tasks.

Expert	Task	Comment
P1	Task-1	Loading files is very immediate, which is not common in the similar tools I used before.
P2	Task-3	It is straightforward for users to have an overview of the whole application.
P4	Task-3	It is considerably more convenient to simultaneously check both 2D and 3D visualizations in the same panel. Putting reconstruction as the first module is valuable for domain users to better understand the problems.
P2, P3	Task-4	The segmentation methods are diverse, and selection is easy.
P1	Task-5	It’s very time-saving to observe the histograms of the images as they took only a short time to be displayed.
P2	Task-7	ERT-Vis possesses a consistent and coherent workflow which makes it comfortable for users to follow. It was advisable to implement it in real time experiments.
P3	Task-8	Amazed by the content contained in a single application as it supports multi-modal visual analysis.
P1	Task-1	Loading files is very immediate, which is not common in the similar tools I used before.
P2	Task-3	It is straightforward for users to have an overview of the whole application.

## Data Availability

Data available in a publicly accessible repository with https://doi.org/10.34658/RDB.UUOQVD (accessed on 30 April 2022).
